# NUF2 Is a Potential Immunological and Prognostic Marker for Non-Small-Cell Lung Cancer

**DOI:** 10.1155/2022/1161931

**Published:** 2022-05-12

**Authors:** Xia Li, Lianlian Zhang, Zhongquan Yi, Jing Zhou, Wenchun Song, Panwen Zhao, Jixiang Wu, Jianxiang Song, Qinggan Ni

**Affiliations:** ^1^Department of General Medicine, The Sixth Affiliated Hospital of Nantong University, Yancheng Third People's Hospital, Jiangsu Province, China; ^2^The Central Laboratory, The Sixth Affiliated Hospital of Nantong University, Yancheng Third People's Hospital, Jiangsu Province, China; ^3^Department of Ultrasound Imaging, The Fourth Affiliated Hospital of Nantong University, Yancheng First People's Hospital, Jiangsu Province, China; ^4^Department of Thoracic Surgery, The Sixth Affiliated Hospital of Nantong University, Yancheng Third People's Hospital, Jiangsu Province, China; ^5^Department of Burns and Plastic Surgery, The Fourth Affiliated Hospital of Nantong University, Yancheng First People's Hospital, Jiangsu Province, China

## Abstract

**Background:**

Globally, non-small-cell lung cancer (NSCLC) is one of the most prevalent tumors. Various studies have investigated its etiology, but the molecular mechanism of NSCLC has not been elucidated.

**Methods:**

The GSE19804, GSE118370, GSE19188, GSE27262, and GSE33532 microarray datasets were obtained from the Gene Expression Omnibus (GEO) database for the identification of genes involved in NSCLC development as well as progression. Then, the identified differentially expressed genes (DEGs) were subjected to functional enrichment analyses. The protein-protein interaction (PPI) network was built after which module analysis was conducted via the Search Tool for Retrieval of Interacting Genes/Proteins (STRING) and Cytoscape. There were 562 DEGs: 98 downregulated genes and 464 upregulated. These DEGs were established to be enriched in p53 signaling pathway, transendothelial leukocyte migration, cell adhesion molecules, contractions of vascular smooth muscles, coagulation and complement cascades, and axon guidance. Assessment of tumor immunity was performed to determine the roles of hub genes.

**Results:**

There were 562 dysregulated genes, while 12 genes were hub genes. NUF2 was established to be a candidate immunotherapeutic target with potential clinical implications. The 12 hub genes were highly enriched in the p53 signaling pathway, the cell cycle, progesterone-associated oocyte maturation, cellular senescence, and oocyte meiosis. Survival analysis showed that NUF2 is associated with NSCLC occurrence, invasion, and recurrence.

**Conclusion:**

The NUF2 gene discovered in this study helps us clarify the pathomechanisms of NSCLC occurrence as well as progression and provides a potential diagnostic and therapeutic target for NSCLC.

## 1. Background

Due to the increase in personal stress, lifestyle changes, the decline in environmental quality, exposure to secondhand tobacco smoke, and a series of other reasons, the incidence of tumors is high, and NSCLC is a very prevalent tumor type [1]. The NSCLC-associated mortality rate is among the highest among all malignancies, and its 5-year survival rate is low, relative to that of other tumors [2]. The development of NSCLC is a great burden to patients and their families. Thus, is it important to determine how to reduce the incidence of NSCLC. First, maintaining a healthy lifestyle is important, and second, high-quality and precise treatment methods are essential. The development and progression of NSCLC are linked to various factors, including genetic aberrations and immune infiltration [3]. Despite extensive studies on the pathomechanisms of its occurrence and progression, the clinical etiology of NSCLC is unclear [4]. Through bioinformatics analysis tools and major database data, we can efficiently search for a target to combat tumors and achieve early detection and prompt intervention in the early stages of tumors to avoid further development of tumors [5].

The histological forms of NSCLC are lung adenocarcinoma (LUAD), large cell carcinoma, and lung squamous cell carcinoma (LUSC). Its development is multistep, with abnormal gene expression as the main feature; this aberrant gene expression leads to phenotypic cell transformation [6–8]. Genetic changes within the genome have been evaluated by ribonucleic acid sequencing (RNA-Seq) [9]. Compared to traditional methods, systematic and comprehensive studies of interactions between differentially enriched pathways and protein-coding genes can precisely establish the carcinogenic effects of changes that occur in the course of NSCLC progression and development. Thus, the analysis of RNA-Seq data using bioinformatics tools can help us understand the pathomechanisms and identify important tumor biomarkers [10]. RNA-Seq is important for identification of key genes that play important roles in disease progression that may help clarify gene expression variations that occur in the course of NSCLC progression. To date, the principal driving force for carcinogenesis is still unclear, which limits the development of NSCLC-targeted therapy [11–14]. Thus, elucidation of NSCLC pathogenesis is still a major challenge, with various key genes yet to be established.

Current microarray technologies and biotin morphology analysis have begun to approach this scope of coverage in almost all tumors. Their applications in screening key gene changes have helped us identify the carcinogenesis-related functions of DEGs and the pathways that are activated in the development of NSCLC [15]. Nevertheless, the true positive rate in independent microarray analysis is not very high, so there are often false positives or false negatives. Therefore, to decrease the false positive rate, we chose five gene sets (GSE19804 [16], GSE118370 [17], GSE19188 [18], GSE27262 [19], and GSE33532 [20]). Then, we used R package from the Bioconductor project [21] and Venn's “LIMMA” graphic software to acquire sets of DEGs between tumor and normal samples in the above five datasets. Third, the Database for Annotation, visualization and comprehensive Discovery (DAVID) was used. Enrichment analysis of the DEGs revealed their related molecular functions (MFs), cell components (CCs), and biological processes (BPs) as well as the Kyoto Protocol Encyclopedia of Genes and Genomes (KEGG) pathways. The protein-protein interaction (PPI) network was built, after which cellular molecular complexity detection (MCODE) was performed to determine various important modules. MCODE was also used for screening 12 hub genes. To obtain vital prognostic data, the dominant genes were imported into the online Kaplan-Meier plotter database (*P* < 0.05). The levels of DEGs and hub genes in NSCLC as well as normal lung tissues were verified by Gene Expression Profiling Interactive Analysis (GEPIA; *P* < 0.05). Overall, the goal of this research was to improve the understanding of the carcinogenic effects of NSCLC through the analysis of data about the processes of genetic variations that occur during disease development and reveal central genes that can be used as biomarkers for diagnosis, therapeutic outcomes, and disease progression.

## 2. Results

### 2.1. NSCLC-Associated DEGs

**Figure 1:**
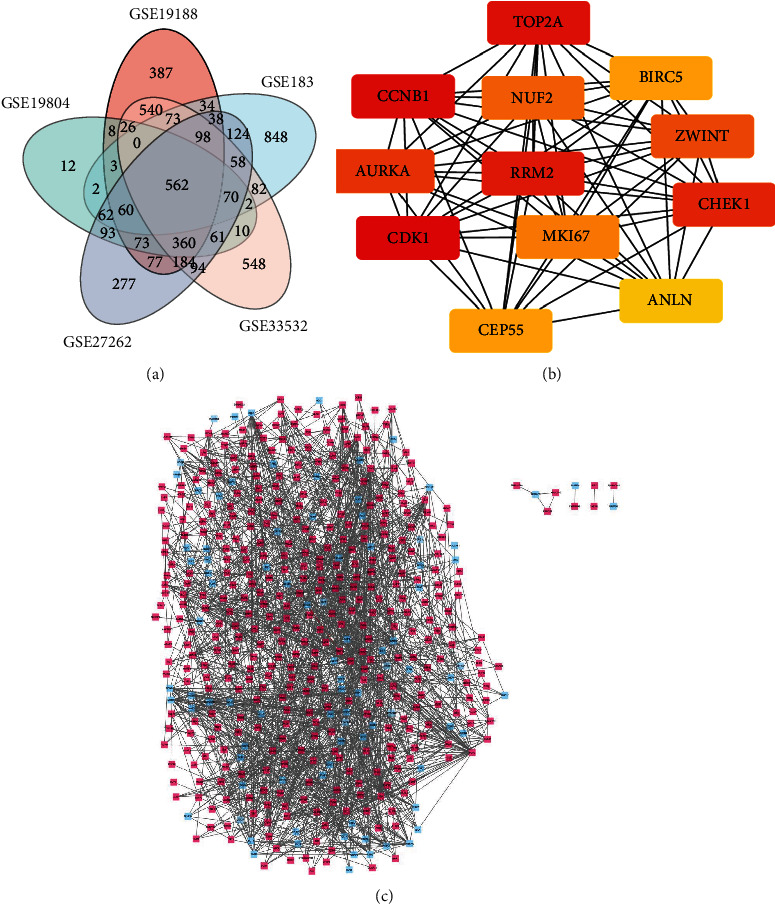
Venn diagram, PPI network, and the most significant module of DEGs. (a) DEGs were selected with a fold change > 1 and *P* value < 0.01 among the mRNA expression profiling set GSE19804, GSE118370, GSE19188, GSE27262, and GSE33532. The 5 datasets showed an overlap of 562 genes. (b) The most significant module was obtained from PPI network with 12 nodes and 66 edges. (c) The PPI network of DEGs was constructed using Cytoscape. Upregulated genes are marked in light red; downregulated genes are marked in light blue.

There were 562 DEGs in the 5 datasets, which consisted of 98 downregulated and 464 upregulated genes (Figure 1(a)).

### 2.2. KEGG and Gene Ontology (GO) Enrichment Analyses

GO analysis revealed that the DEGs were markedly enriched in BPs, such as extracellular matrix organization, extracellular structure organization, nuclear division, mitotic nuclear division, organelle fission, cell-substrate adhesion, assembly of cell junctions, organization of cell junctions, vascular process in circulatory system, and ameboidal-type cell migration (Table 1). The enriched MFs included actin binding, extracellular constituents of matrix structures, amyloid-beta binding, peptide binding, amide binding, histone kinase activity, metalloendopeptidase activity, extracellular constituents of matrix structures conferring tensile strengths, and metallopeptidase activities (Table 1). The enriched CC terms were cell-cell junction, actin filament bundle, stress fiber, contractile actin filament bundle, contractile fiber part, midbody, condensed chromosome, centromeric region, chromosomal region, and condensed nuclear chromosome (Table 1). KEGG pathway analyses showed that downregulated DEGs were highly enriched in p53 signaling pathway, while upregulated DEGs were highly enriched in pathways related to cell adhesion molecules, transendothelial leukocyte migration, contractions of vascular smooth muscles, coagulation and complement cascades, and axon guidance.

### 2.3. The PPI Network and Module Analysis

The established DEG-associated PPI network is shown in Figure 1(c), with the most important module shown in Figure 1(b), as identified by Cytoscape. Functional assessments of genes in this module revealed high enrichment in nuclear division, organelle fission, mitotic nuclear division, histone phosphorylation, cell cycle checkpoint, condensed chromosome, centromeric region, chromosomal region, midbody, chromosomes, protein serine/threonine kinase activities, histone kinase activities, protein C-terminus binding, the cell cycle, progesterone-mediated oocyte maturation, ferric iron binding, oxidoreductase activities, acting on CH or CH2 groups, the p53 signaling pathway, cellular senescence, and oocyte meiosis (Table 2).

### 2.4. Hub Gene Identification and Analysis

**Figure 2:**
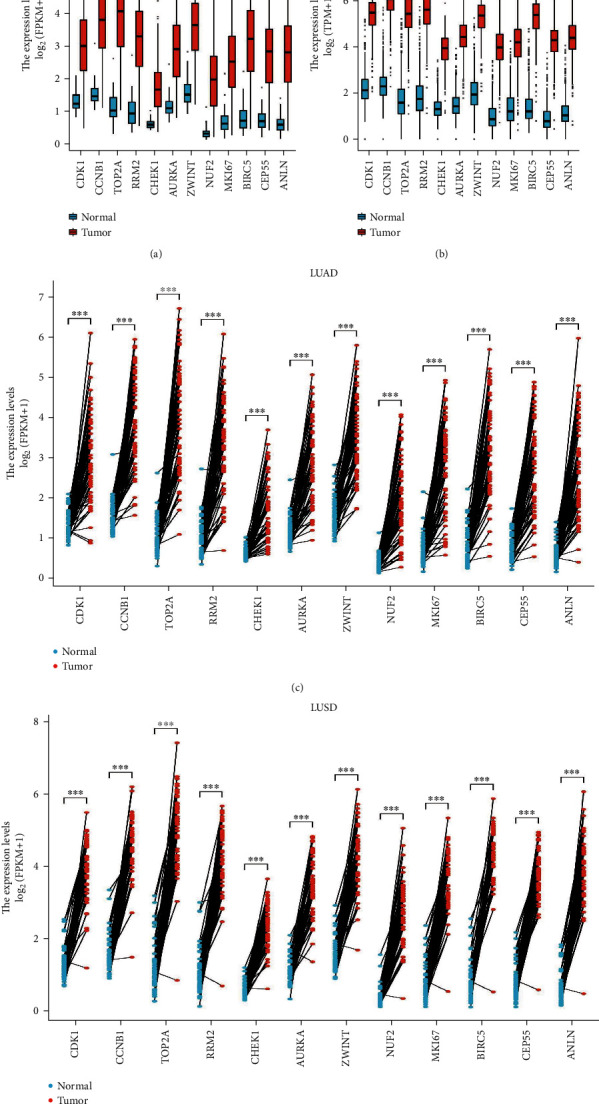
The 12 hub genes were used to draw the difference in the distribution of lung adenocarcinoma and lung squamous cell carcinoma tissues and adjacent tissues in the TCGA (https://portal.gdc.cancer.gov/) database using ggplot2 in R language. (a) is the expression of hub gene in cancer tissues and adjacent tissues of unpaired samples in LUAD, and (b) is the expression of hub gene in cancer tissues and adjacent tissues of unpaired samples in LUSD. (c) represents the expression of hub gene in LUAD paired sample cancer tissues and adjacent tissues, and (d) represents the expression of hub gene in LUSD paired sample cancer tissues and adjacent tissues. The *P* value adopts scientific notation and all have statistical significance. ^∗^*P* < 0.05; ^∗∗^*P* < 0.01; and ^∗∗∗^*P* < 0.001.

**Figure 3:**
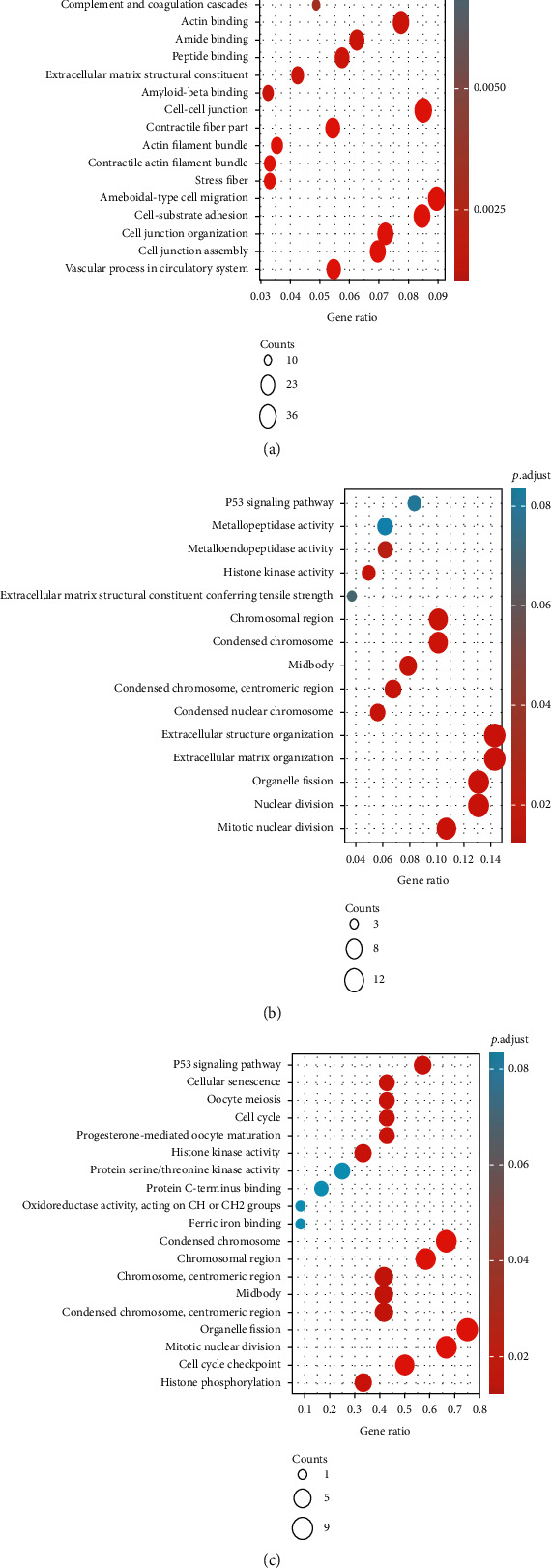
Download the hub gene from the DAIVED website (https://david.ncifcrf.gov/), and then, use the ggplot2 package and clusterProfiler package in the R language to visualize the enrichment analysis of GO and KEGG. Panel (a) is the enrichment analysis of upregulated genes, and panel (b) is the enrichment analysis of downregulated genes. Panel (c) is the visualization diagram of the enrichment analysis of the hub gene.

**Figure 4:**
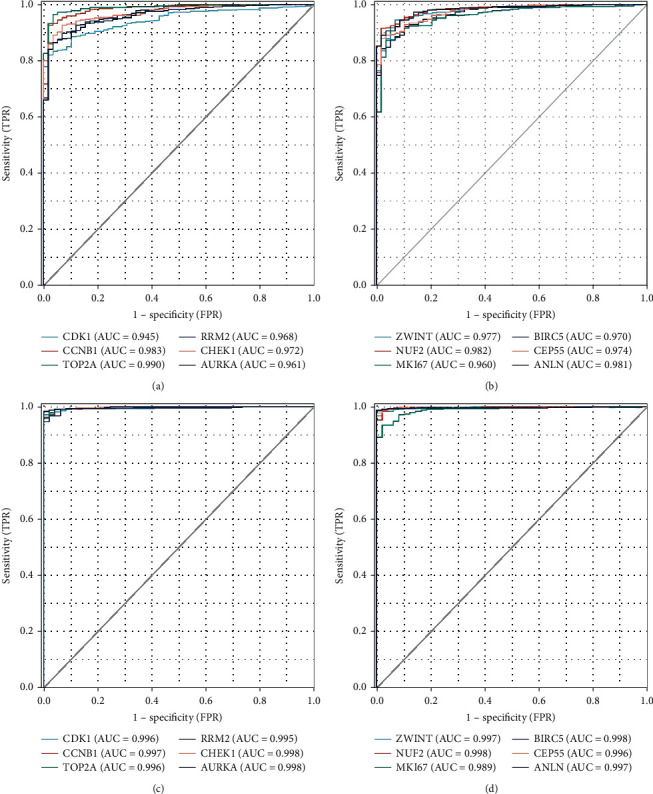
The 12 hub genes were analyzed with the pROC package in R language and visualized with the ggplot2 package. (a) and (b) are receiver operating characteristic curves (ROC) of LUAD, and (c) and (d) are receiver operating characteristic curves (ROC) of LUSC.

**Figure 5:**
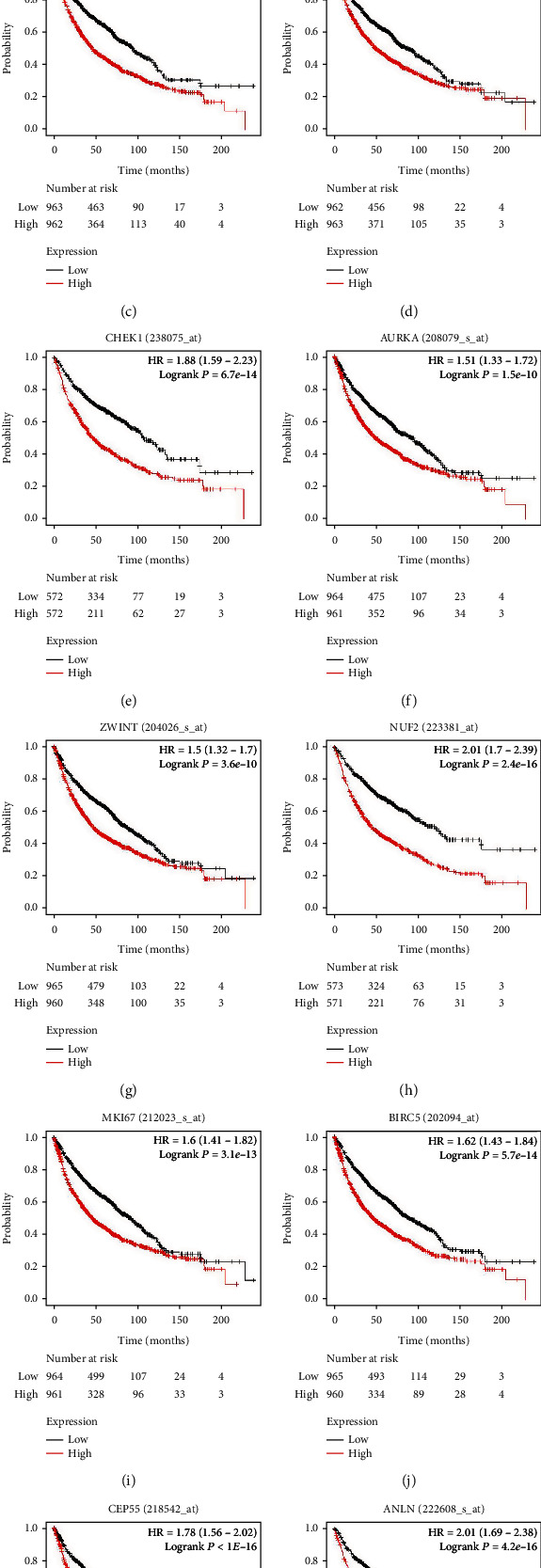
Overall survival analyses of 12 hub genes were performed using the Kaplan-Meier plotter online platform. *P* < 0.05 was considered statistically significant.

**Figure 6:**
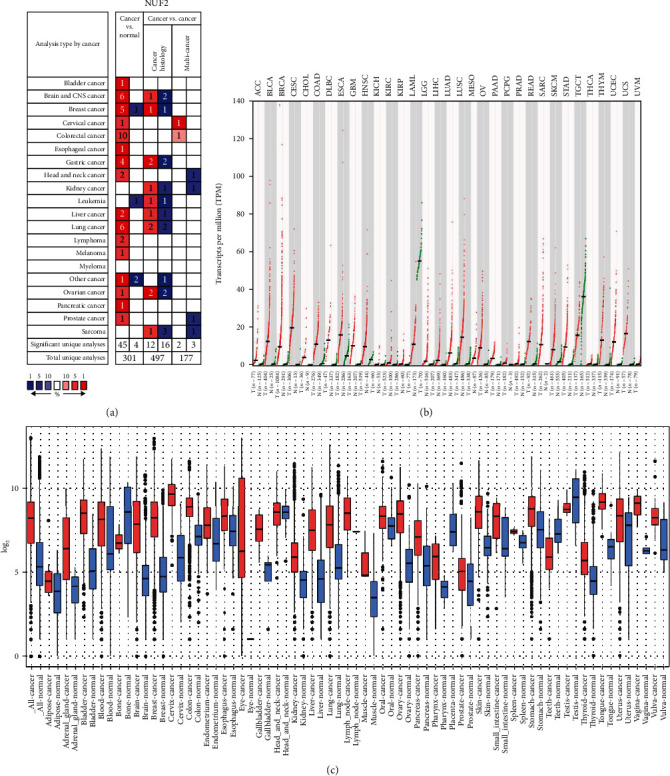
NUF2 mRNA expression in a variety of cancer types: (a) Comparison shows that the datasets of NUF2 mRNA overexpression (left column, red) and underexpression (right column, blue) are in cancer and normal tissues. The graphic representation is derived from the Oncomine database (available from https://www.oncomine.org/resource/login.html), and the threshold is to use the following parameters: *P* value is 1E-4, fold change is 2, and gene ranking is 10%. (b) Expression of NUF2 expression in 33 human cancers (Gene Expression Profiling Interactive Analysis 2) (URL https://gepia2.cancer-pku.cn) obtained from the Cancer Genome Atlas via GEPIA2: dot map. The gene expression profiles in all tumor samples and paired normal tissues are shown. Each point represents the expression of the sample. (c) The expression pattern of NUF2 mRNA in tumors and tissues. The corresponding normal tissues: the data on the expression of NUF2 mRNA in various types of cancer is retrieved from the GENT (gene expression in normal and tumor tissue) database (available at http://medicalgenomics.kribb.re.kr/GENT/). The boxes represent the median and the 25th and 75th percentiles. Dots represent outliers. The red box represents tumor tissue, and the blue box represents normal tissue.

Twelve genes were established to be hub genes with degree values ≥ 10 (Table 3). The 12 hub genes were used to draw the difference in the distribution of LUAD and LUSC tissues and adjacent tissues in TCGA database (https://portal.gdc.cancer.gov/) using ggplot2 in R language.  Figure 2(a) shows the expression levels of hub genes in unpaired LUAD as well as adjacent tissues. The levels of hub genes in unpaired LUAD and adjacent tissues are shown in Figure 2(b).  Figure 2(c) shows the levels of hub genes in paired LUAD tumor and adjacent tissues.  Figure 2(d) shows the levels of hub genes in adjacent and paired LUSC tissues. The *P* value was used to indicate significance as follows: ns, *P* ≥ 0.05; ∗, *P* < 0.05; ∗∗, *P* < 0.01; and ∗∗∗, *P* < 0.001. Hub genes were downloaded from the DAVID website (https://david.ncifcrf.gov/), and then, the “ggplot2” and “clusterProfiler” in R language were used for visualization the GO and KEGG results. Enrichments of upregulated and downregulated genes are shown in Figures 3(a) and 3(b), respectively.  Figure 3(c) shows a visualization of the enrichment analysis results for the hub genes. After the analysis of the 12 hub genes with the pROC package in R language and visualized with the ggplot2 package, receiver operating characteristic (ROC) curves were generated for LUAD (Figures 4(a) and 4(b)) and LUSC (Figures 4(c) and 4(d)). NUF2 assessment showed higher accuracy than other variables in predicting the tumor status (normal versus tumor). Subsequently, overall survival analysis according to hub gene expression was conducted via the Kaplan-Meier curves. Relative to low expression patients, NSCLC patients with elevated CDK1, CCNB1, TOP2A, PRM2, CHEK1, AURKA, ZWINT, NUF2, MKI67, BIRC5, CEP55, and ANLN levels showed worse overall survival (Figures 5(a)–5(h)). We noticed that NSCLC patients with changes of NUF2-related genes showed decreased overall survival, while NSCLC patients with NUF2 genome changes showed the highest hazard ratio. These observations were statistically significant (HR = 2.01, CI 1.7-2.39, *P* = 2.4e − 16) (Figure 5(h)). Assessment of NUF2 mRNA levels in a variety of tumor types revealed that they were elevated in tumor tissues, relative to adjacent tissues (Figure 6(a)). Comparison analysis showed that NUF2 mRNA is high (left column, red) and suppressed (right column, blue) in tumor and normal tissues, respectively. The diagram comes from the Oncomine database (available from https://www.oncomine.org/resource/login.html) with thresholds as: *P* value, 1E-4; fold change, 2; and gene rank, 10%.  Figure 6(b) shows the expression of NUF2 in 33 human cancer datasets (GEPIA2) (URL https://gepia2.cancer-pku.cn) obtained from the TCGA via GEPIA2; dot plots were generated to show all the gene expression profiles. The cancer and their matching normal tissues were collected. Each point represents the expression of the sample.  Figure 6(c) shows NUF2 mRNA levels in tumors and tissues. Data for adjacent normal tissues were acquired from the Gene Expression in Normal and Tumor Tissues (GENT) database (http://medicalgenomics.kribb.re.kr/GENT/). The boxes denote the median as well as the 25^th^ and 75^th^ percentiles. Dots denote outliers. The red box represents cancer tissue, and the blue box represents normal tissue.

**Figure 7:**
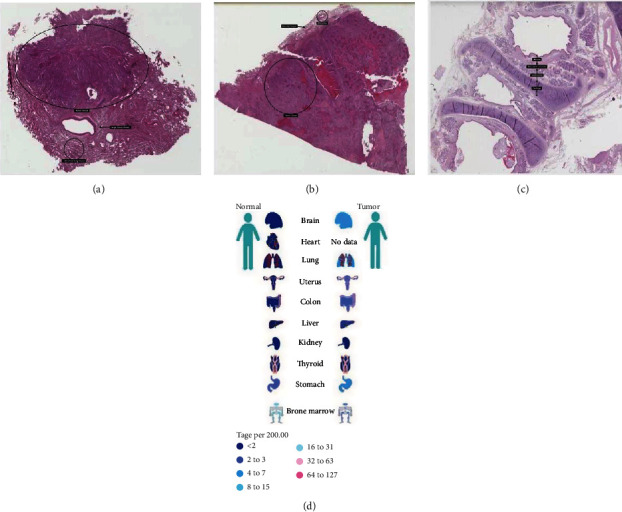
Use Protein Atlas network database (https://www.proteinatlas.org/) to analyze: (a) is the HE staining result of NUF2 in lung adenocarcinoma, and (b) is the HE staining result of NUF2 in lung squamous cell carcinoma. (c) is the HE staining result of NUF2 in normal bronchus. (d) is the use of SAGE to analyze NUF2 in human cancers.

**Figure 8:**
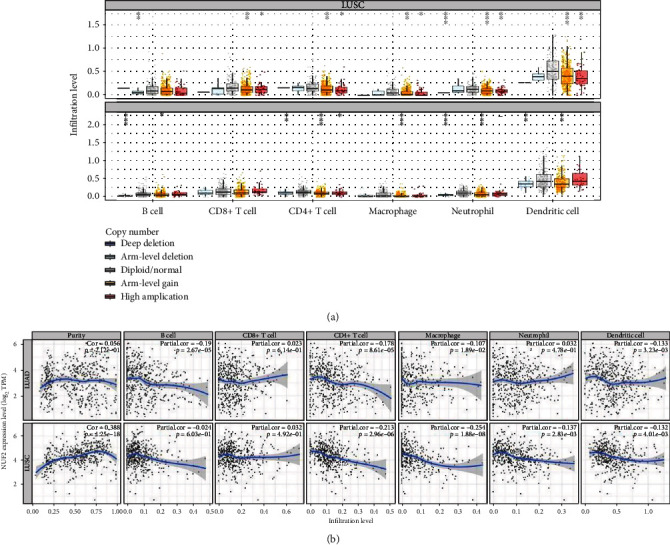
(a) Use the TIMER website (https://cistrome.shinyapps.io/timer/) to explore the correlation between tumor immune cell infiltration and somatic cell copy number of NUF2 in LUAD and LUSD, respectively. According to the copy number of NUF2, samples are divided into five categories (deep deletion, arm-level deletion, diploid/normal, arm-level gain, and high amplication); compare the distribution of immune cells infiltrated among the five types of samples. *P* value significant codes: ^∗∗∗^*P* < 0.001, ^∗∗^*P* < 0.01, and ^∗^*P* < 0.05. (b) Use the TIMER website (https://cistrome.shinyapps.io/timer/) to view the correlation between immune cell and tumor purity and NUF2 expression in LUAD and LUSD in the TCGA database.

**Figure 9:**
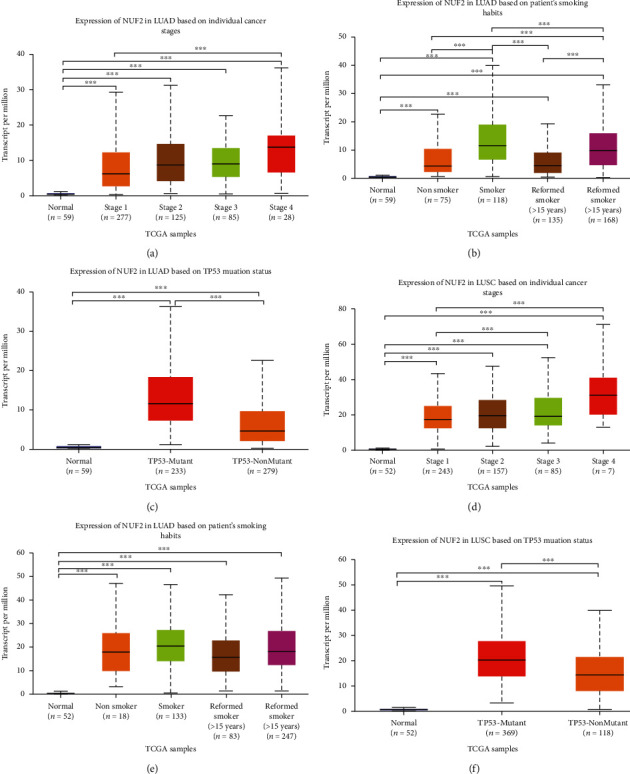
(a–c) Use the UALCAN website (http://ualcan.path.uab.edu/cgi-bin/ualcan-res.pl), respectively, to show the expression differences of NUF2 lung adenocarcinoma tumors in different grades, normal population, and lung adenocarcinoma. The expression of NUF2 in different smokers and the difference in the expression of NUF2 in TP53 mutant and nonmutated lung adenocarcinoma. (d–f) The differences in the expression of NUF2 lung squamous cell carcinoma tumors of different grades, the expression of NUF2 in normal people and different smokers of lung squamous cell carcinoma, and the difference in expression of NUF2 in TP53 mutated and nonmutated lung squamous cell carcinoma. ^∗∗∗∗^*P* < 0.001 indicates that the difference is statistically significant.

The Protein Atlas network database (https://www.proteinatlas.org/) was utilized for analyses.  Figure 7(a) shows the hematoxylin and eosin (HE) staining result for NUF2 in LUAD, and Figure 7(b) shows the HE staining result for NUF2 in LUSC.  Figure 7(c) shows the HE staining result for NUF2 in normal bronchial tissues.  Figure 7(d) shows the Serial Analysis of Gene Expression (SAGE) findings for the analysis of NUF2 in human cancers. The Tumor IMmune Estimation Resource (TIMER) website (https://cistrome.shinyapps.io/timer/) was used to show the associations between infiltrations of immune cells in tumors and NUF2 somatic copy numbers in LUSC and LUAD. According to the copy number of NUF2, the samples were divided into five categories (arm-level deletion, deep deletion, diploid/normal, high amplification, and arm-level gain), and distributions of infiltrated immune cells in the five samples were compared. In LUAD, except for the marked variations in the number of arm-level deletions in B cells, the other five types of cells (neutrophils, CD8+ T cells, macrophages, CD4+ T cells, and dendritic cells) were diploid/normal or showed significant differences in arm-level gain. However, the results were not exactly the same for LUSC. The copy numbers in neutrophils, B cells, and dendritic cells showed obvious differences. The rates of arm-level deletions as well as gains and increased amplification in CD4+ T cells showed obvious differences. The arm-level gain in macrophages was obviously different, and all CD8+ T cell copy number observations were significantly different between normal and tumor tissues. *P* values are as follows: ns, *P* ≥ 0.05; ∗, *P* < 0.05; ∗∗, *P* < 0.01; and ∗∗∗, *P* < 0.001 (Figure 8(a)). The TIMER website (https://cistrome.shinyapps.io/timer/) was used to view the correlations of immune cells and tumor purity and NUF2 levels in LUSC and LUAD in the TCGA database (Figure 8(b)).  Table 4 shows the correlation analysis of NUF2 and immune cell-associated genes as well as biomarkers in TIMER (*P* ≤ 0.001). The UALCAN website (http://ualcan.path.uab.edu/cgi-bin/ualcan-res.pl) was used to show differences in the levels of NUF2 in LUAD cancers of different grades and in the normal population versus LUAD patients. The expression of NUF2 in patients with different smoking statuses and the difference in the expression of NUF2 in TP53 mutant and nonmutated LUAD were assessed (Figures 9(a)–9(c)). The differences in the expression of NUF2 in LUSC tumors of different grades, the expression of NUF2 in normal people and LUSC patients with different smoking statuses, and the difference in the expression of NUF2 in TP53-mutated and nonmutated LUSC samples were also assessed. ^∗∗∗^*P* < 0.001 indicates that the difference is statistically significant (Figures 9(d)–9(f)).

### 2.5. NUF2 Expression in NSCLC Tissues

**Figure 10:**
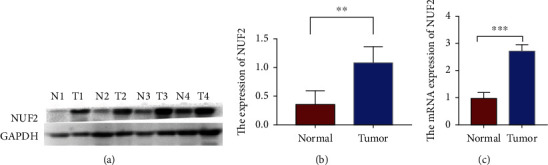
(a and b) Expression of NUF2 in four non-small-cell lung cancer tissues and adjacent normal tissues detected by western blotting. GADPH was used as loading control. ^∗^*P* < 0.05 and ^∗∗^*P* < 0.01. (c) The mRNA expression of NUF2 in four non-small-cell lung cancer tissues and adjacent normal tissues detected by RT-PCR.

To evaluate the significance of NUF2 in NSCLC, we investigated NUF2 protein levels in four randomly selected paired NSCLC specimens. Western blot showed elevated NUF2 levels in NSCLC tissues, relative to adjacent nontumor samples (Figures 10(a) and 10(b), *P* < 0.01).

## 3. Discussion

Globally, lung carcinoma is the most prevalent cause of tumor-associated death. About 1.6 million people die from lung carcinoma each [22]; approximately 85% of lung cancer patients have NSCLC, among which LUAD and LUSC are the most prevalent subtypes [23]. With continuous advances in molecular biology and information technology, in the past two decades, significant progress in NSCLC treatment has been reported [24]. Smoking is highly correlated with the development of lung carcinoma, and it is also related to environmental exposures, such as secondhand smoke, occupational carcinogens and pollution, and genetic susceptibility [25, 26]. However, the pathomechanisms for NSCLC occurrence as well as development of NSCLC are not extremely clear. Regulators of the cell cycle play major roles in NSCLC [27–29]. Most NSCLC cases are not detected early, making patients ineligible for treatment, which could explain for poor prognostic outcomes. Thus, the need for development of potential markers for efficient diagnosis and treatment is urgent. Microarray technologies allow the exploration of genetic changes in NSCLC and have been proven to be important methods for identifying new disease markers [30].

In this study, the pathways in which the DEGs were found to be enriched are closely related to immune infiltration. Mami-Chouaib et al. studied resident memory T cells and found that they are critical components in tumor immunology [31]. The tumor microenvironment (TME) affects the progression of many malignant tumors in humans. The infiltration of immune-related cells into tumors increases the recruitment of immune activation signals and antidisease immune effector cells and activates related pathways [32]. KEGG pathway analyses showed that downregulated DEGs were highly enriched in p53 signaling pathway. The p53 protein can mediate nucleolar stress responses, leading to cell cycle arrest, apoptosis, senescence, or differentiation, thereby affecting the occurrence as well as development of tumors [33]. Mutations of the p53 tumor suppressor gene often occur in lung carcinoma. Mutant p53 (mtp53) suppresses wild-type p53 protein activities and destroy its tumor suppressor function. Moreover, mtp53 usually functions as an oncogene. The posttranslational modification of p53 protein is vital for its transcription as well as tumor suppressor function [34]. These conclusions are in tandem with ours.

Twelve DEGs with degrees ≥ 10 were obtained as hub genes. We noticed that NSCLC patients with NUF2-related genomic changes showed a decrease in overall survival, while NSCLC patients with NUF2 genome changes showed the highest hazard ratio. NUF2 is a component of the essential kinetochore-associated NDC80 complex, which is important in chromosomal segregation as well as spindle checkpoint activities. It is also vital for the maintenance of the integrity of the kinetochore and organization of stable microtubule binding sites in the outer plate of the kinetochore. The complex promotes the affinity of the SKA1 complex for microtubules, which allows the NDC80 complex to track depolymerizing microtubules [35]. NUF2 is reported as one of tumor testis antigens that is secreted ectopically by cancers, and NUF2 levels are increased in prostate tumor tissues [36]. Xie et al. found that NUF2 is involved in cell apoptosis and proliferation regulation by controlling the binding of spindle microtubules and the centromere to attain the correct chromosome separation. NUF2, a prognostic-associated marker, is correlated with infiltrations of immune cells in hepatocellular carcinoma [37]. In addition, NUF2 is elevated in breast cancer, human osteosarcoma, pancreatic tumor, and colorectal cancer and is an important diagnostic, treatment, and prognostic marker of tumors [38–41]. In conclusion, NUF2 is a potential predictor of NSCLC prognosis.

The TME is a key regulator of tumorigenesis, tumor progression, and drug resistance [42]. In the TME, tumors and cells continue to evolve to reduce the production of new antigens and the burden of mutations to facilitate the evasion of antitumor responses. This reduces the tumor's responsiveness to adaptive immune responses and facilitates cancer-supportive changes inside the tumor, such as changes in the expression of immunomodulatory molecules on cancer cells. External tumor factors, including soluble inhibitory molecules, immunosuppressive cells, or inhibitory receptors expressed by immune cells, can change the compositions and activities of tumor-infiltrating lymphocytes (TILs) (by enhancing the T regulatory cell, effector T cell ratio, and suppressing the roles of effector T cells and enhancing tumor proliferation as well as metastasis) [43]. We found that NUF2 is associated with immune infiltration of several cell types, such as CD8+ T cells, B cells, T cells (general), tumor-associated macrophages (TAMs), monocytes, M1 and M2 macrophages, natural killer cells, neutrophils, dendritic cells, and Th1, Th2, Tfh, Th17, Treg, and exhausted T cells. The association between NUF2 and immunosuppressive gene levels implies that NUF2 has a major function in regulation of cancer immunology.

In summary, this study was aimed at identifying DEGs that play key roles in NSCLC occurrence or progression. There were 562 DEGs and 12 hub genes, and these genes can be used as diagnostic markers for NSCLC. These results also prove that NUF2 can be used as an effective immunotherapy target. In the next step, our research group will use molecular biology experiments to further verify the biological functions of NUF2 in NSCLC in vivo and in vitro. Finally, we will use western blotting to evaluate the levels of NUF2 in NSCLC and adjacent tissues to verify that NUF2 can indeed be used as a target for in-depth research on NSCLC treatment.

## 4. Methods

### 4.1. Microarray Data

The Gene Expression Omnibus (GEO) (http://www.ncbi.nlm.nih.gov/geo) [44] is a public functional genomics data repository of high-throughput gene expression and chip and microarray data. Five gene expression datasets (GSE19804 [16], GSE118370 [17], GSE19188 [18], GSE27262 [19], and GSE33532 [20]) were retrieved from the GEO (GPL570 Platform Affymetrix Human Genome U133 Plus 2.0 Array). The conversion of probes into their corresponding gene symbols was based on annotation information for the platform. The GSE19804 dataset had 60 NSCLC tissue and 60 noncancerous samples. The GSE118370 dataset had 6 NSCLC tissue and 6 noncancerous samples. The GSE19188 dataset had 91 NSCLC tissue and 65 noncancerous samples. The GSE27262 dataset had 25 NSCLC tissue and 25 noncancerous samples. The GSE33532 dataset had 80 NSCLC tissue and 20 noncancerous samples.

### 4.2. Identification DEGS

GEO2R (http://www.ncbi.nlm.nih.gov/geo/geo2r) was used for screening DEGs between NSCLC samples and noncancer samples. GEO2R, an interactive web tool, enables the comparisons of two or more GEO datasets. To identify DEGs, we applied thresholds for the adjusted (adj.) *P* value and Benjamini and Hochberg false discovery rate to establish a balance between the limitations of finding significant (statistical) genes as well as false positives. Probe sets lacking the corresponding gene symbols or genes exhibiting multiple probe sets were, respectively, eliminated or averaged. Log fold change (FC) > 1 and adj. *P* < 0.01 denoted statistical significance [45].

### 4.3. KEGG and GO Analyses of the DEGs

DAVID (http://david.ncifcrf.gov) (version6.7) [46] is an online biological information database integrated with a comprehensive set of analysis tools. Functional annotation of genes and proteins can be used to extract biological information. KEGG is a database resource for understanding advanced and biological functions. Systems generated from large-scale molecular datasets are considered high-throughput experimental techniques [47] GO is an established gene analysis method.

### 4.4. PPI Network Construction and Module Analysis

The Search Tool for the Retrieval of Interacting Genes (STRING; http://string-db.org) (version 10.0) [48] online database was used for PPI network prediction. Analysis of functional interactions between and among proteins may elucidate on the pathomechanisms of various diseases. We used the STRING database to build a PPI network of DEGs, and interactions with a combined score > 0.4 were considered statistically significant. Cytoscape (version 3.4.0) is an open source bioinformatics software platform for visualizing molecular interaction networks [49]. The MCODE (version 1.4.2) plug-in of Cytoscape is an app for clustering a given network based on topology to find densely connected regions [50]. The PPI networks were drawn using Cytoscape, with the most significant module in the networks identified using MCODE. The selection criteria were MCODE score > 5, degree cutoff = 2, node score cutoff = 0.2, max depth = 100, and *k* − score = 2. Then, KEGG and GO analyses of the genes in this module were conducted using DAVID.

### 4.5. Hub Gene Selection and Analysis

Hub genes with degrees ≥ 10 were selected for analysis. A network of the genes and their coexpressed genes was analyzed using cBioPortal (http://www.cbioportal.org) [51, 52] online platform. The biological process analysis of hub genes was performed and visualized using the Biological Networks Gene Oncology (BiNGO) (version 3.0.3) plugin of Cytoscape [53]. Hierarchical clustering of hub genes was performed using the UCSC Cancer Genomics Browser (http://genome-cancer.ucsc.edu) [54]. Overall survival and disease-free survival analyses of hub genes were performed using the Kaplan-Meier curves in cBioPortal. The expression profiles of NUF2 were analyzed and displayed using the online database SAGE (http://www. http://ncbi.nlm.nih.gov/SAGE). The relationships between expression patterns and tumor grades, infection status, metastasis, and vascular invasion were analyzed using the online database Oncomine (http://www.oncomine.com) [55–57].

### 4.6. NSCLC Patient Specimens

To investigate NUF2 levels in human NSCLC, we obtained tumor tissues and paired adjacent nontumorous tissues during radical resection of patients without prior chemotherapy or radiotherapy at the Department of Thoracic Surgery, Sixth Affiliated Hospital of Nantong University. Resected NSCLC-adjacent nontumor samples and matched tumor tissues were obtained and instantly stored in liquid nitrogen (Table 5). From May 2021 to July 2021, 2 pairs of lung squamous cell carcinoma tissues and adjacent nontumor tissues and 2 pairs of lung adenocarcinoma tissues and adjacent nontumor tissues were randomly selected from patients in The Sixth Affiliated Hospital of Nantong University (Yancheng Third People's Hospital) (T1 and T2 in Figure 10 are LUSCs, and T3 and T4 are LUADs). All patients or their guardians provided informed consents, and this study was approved by the ethical committee of The Sixth Affiliated Hospital of Nantong University (Yancheng, China). The grade and histological type of all tissue samples were independently verified by two professional pathologists.

### 4.7. Western Blot Analysis

A lysis buffer (Beyotime Institute of Biotechnology, Nantong, China) was used to prepare total protein extracts from cell lines as well as tumor tissues. Then, protein concentrations were evaluated by a BCA kit (Beyotime Institute of Biotechnology, Nantong, China). An equal protein amount (40 *μ*g per lane) was separated by SDS-polyacrylamide gel electrophoresis (PAGE) in 12% acrylamide gels and transferred onto polyvinylidine difluoride (PVDF) membranes (Millipore Corporation, Billerica, USA) which were blocked in 5% fat-free milk followed by overnight incubation at 4°C with the rabbit anti-NUF2 primary antibody (1 : 5000 dilution; Sangon Biotech, Shanghai, China). The secondary antibody was horseradish peroxidase- (HRP-) conjugated goat anti-rabbit antibody (1 : 2000; Beyotime Institute of Biotechnology). After stripping, the membrane was reprobed overnight at 4°C with antibody against GAPDH (1 : 2000; Beyotime Institute of Biotechnology), followed by incubation with secondary antibodies as above at room temperature (RT) for 2 h. An enhanced chemiluminescence system (ECL; Beyotime Institute of Biotechnology) was used for band visualization. The band intensities were quantified by densitometry.

### 4.8. Real-Time Quantitative PCR

Quantitative real-time PCR analysis was measured as previously described. Total RNA was isolated from cultured cells or muscle tissues using an RNeasy plus mini kit. cDNA was obtained using a GoScript Reverse Transcription System and analyzed by quantitative real-time PCR using SYBR Green kit. The data were normalized to expression of ribosomal gene NUF2 or GAPDH. The primer sequences are NUF2-forward (ATGGAAGGCTTCTTACCATTCA) and NUF2-reverse (CTTAAAAACCGACTTGTCCGTT).

### 4.9. Statistical Analysis

GraphPad Prism 7.0 software was used for statistical analyses. Between-group differences were evaluated by the two-tailed Student's *t*-test. A *P* value < 0.05 indicated significance. All assays were repeated thrice.

## Figures and Tables

**Table 1:** GO and KEGG pathway enrichment analyses of DEGs in NSCLC samples.

Term	Pathway description	*P* value	Count in gene set
Downregulated			
GO:0030198	Extracellular matrix organization	9.08e-08	12
GO:0043062	Extracellular structure organization	3.96e-07	12
GO:0000280	Nuclear division	2.06e-06	11
GO:0140014	Mitotic nuclear division	2.88e-06	9
GO:0048285	Organelle fission	5.27e-06	11
GO:0000793	Condensed chromosome	7.48e-07	9
GO:0030496	Midbody	1.33e-05	7
GO:0000779	Condensed chromosome, centromeric region	1.57e-05	6
GO:0098687	Chromosomal region	2.82e-05	9
GO:0000794	Condensed nuclear chromosome	8.57e-05	5
GO:0035173	Histone kinase activity	9.26e-07	4
GO:0004222	Metalloendopeptidase activity	1.09e-04	5
GO:0030020	Extracellular matrix structural constituent conferring tensile strength	8.69e-04	3
GO:0008237	Metallopeptidase activity	0.001	5
hsa04115	p53 signaling pathway	8.85e-04	4
Upregulated			
GO:0031589	Cell-substrate adhesion	3.11e-13	34
GO:0034329	Cell junction assembly	4.04e-13	28
GO:0034330	Cell junction organization	6.79e-12	29
GO:0003018	Vascular process in circulatory system	2.33e-11	22
GO:0001667	Ameboidal-type cell migration	2.68e-11	36
GO:0005911	Cell-cell junction	2.24e-11	36
GO:0032432	Actin filament bundle	5.17e-11	15
GO:0001725	Stress fiber	1.23e-10	14
GO:0097517	Contractile actin filament bundle	1.23e-10	14
GO:0044449	Contractile fiber part	4.65e-10	23
GO:0003779	Actin binding	1.49e-08	31
GO:0001540	Amyloid-beta binding	1.93e-08	13
GO:0005201	Extracellular matrix structural constituent	1.76e-07	17
GO:0042277	Peptide binding	2.75e-07	23
GO:0033218	Amide binding	6.00e-07	25
hsa04514	Cell adhesion molecules	2.35e-05	14
hsa04670	Leukocyte transendothelial migration	2.95e-05	12
hsa04270	Vascular smooth muscle contraction	3.57e-05	13
hsa04610	Complement and coagulation cascades	5.31e-05	10
hsa04360	Axon guidance	1.97e-04	14

GO: Gene Ontology; KEGG: Kyoto Encyclopedia of Genes and Genomes; DEGs: differentially expressed genes; NSCLC: non-small-cell lung cancer.

**Table 2:** GO and KEGG pathway enrichment analyses of DEGs in the most significant module.

ID	Description	*P* value	Count in gene set
GO:0000280	Nuclear division	2.12e-13	9
GO:0048285	Organelle fission	5.13e-13	9
GO:0140014	Mitotic nuclear division	6.78e-13	8
GO:0000075	Cell cycle checkpoint	1.95e-09	6
GO:0016572	Histone phosphorylation	7.14e-09	4
GO:0000793	Condensed chromosome	1.12e-13	8
GO:0098687	Chromosomal region	3.76e-10	7
GO:0000779	Condensed chromosome, centromeric region	5.40e-09	5
GO:0030496	Midbody	3.70e-08	5
GO:0000775	Chromosome, centromeric region	6.39e-08	5
GO:0035173	Histone kinase activity	2.87e-10	4
GO:0004674	Protein serine/threonine kinase activity	0.003	3
GO:0008022	Protein C-terminus binding	0.007	2
GO:0008199	Ferric iron binding	0.007	1
GO:0016725	Oxidoreductase activity, acting on CH or CH2 groups	0.009	1
hsa04115	p53 signaling pathway	2.11e-07	4
hsa04914	Progesterone-mediated oocyte maturation	6.22e-05	4
hsa04110	Cell cycle	1.18e-04	4
hsa04114	Oocyte meiosis	1.33e-04	4
hsa04218	Cellular senescence	2.34e-04	4

GO: Gene Ontology; KEGG: Kyoto Encyclopedia of Genes and Genomes; DEGs: differentially expressed genes; FDR: false discovery rate.

**Table 3:** Functional roles of 16 hub genes with degree ≥ 10.

No.	Gene symbol	Full name	Function
1	CDK1	Cyclin-dependent kinase 1	Plays a key role in the control of the eukaryotic cell cycle by modulating the centrosome cycle as well as mitotic onset; promotes G2-M transition, and regulates G1 progress and G1-S transition via association with multiple interphase cyclins. Required in higher cells for entry into S-phase and mitosis
2	CCNB1	G2/mitotic-specific cyclin-B1	Essential for the control of the cell cycle at the G2/M (mitosis) transition; belongs to the cyclin family. Cyclin AB subfamily ZC3HC1-nuclear-interacting partner of ALK; essential component of a SCF-type E3 ligase complex, SCF (NIPA), a complex that controls mitotic entry by mediating ubiquitination and subsequent degradation of cyclin B1 (CCNB1). Its cell-cycle-dependent phosphorylation regulates the assembly of the SCF (NIPA) complex, restricting CCNB1 ubiquitination activity to interphase. Its inactivation results in nuclear accumulation of CCNB1 in interphase and premature mitotic entry. May have an antiapoptotic role in NPM-ALK-mediated signaling events
3	TOP2A	DNA topoisomerase 2-alpha	Control of topological states of DNA by transient breakage and subsequent rejoining of DNA strands. Topoisomerase II makes double-strand breaks. Essential during mitosis and meiosis for proper segregation of daughter chromosomes. May play a role in regulating the period length of ARNTL/BMAL1 transcriptional oscillation (by similarity)
4	PRM2	Ribonucleoside-diphosphate reductase subunit M2	Provides the precursors necessary for DNA synthesis. Catalyzes the biosynthesis of deoxyribonucleotides from the corresponding ribonucleotides. Inhibits Wnt signaling; belongs to the ribonucleoside diphosphate reductase small chain family
5	CHEK1	Serine/threonine-protein kinase Chk1	Serine/threonine-protein kinase which is required for checkpoint-mediated cell cycle arrest and activation of DNA repair in response to the presence of DNA damage or unreplicated DNA. May also negatively regulate cell cycle progression during unperturbed cell cycles. This regulation is achieved by a number of mechanisms that together help to preserve the integrity of the genome.
6	AURKA	Aurora kinase A	Mitotic serine/threonine kinase that contributes to the regulation of cell cycle progression. Associates with the centrosome and the spindle microtubules during mitosis and plays a critical role in various mitotic events including the establishment of mitotic spindle, centrosome duplication, centrosome separation as well as maturation, chromosomal alignment, spindle assembly checkpoint, and cytokinesis. Required for initial activation of CDK1 at centrosomes
7	ZWINT	ZW10 interactor	Part of the MIS12 complex, which is required for kinetochore formation and spindle checkpoint activity. Required to target ZW10 to the kinetochore at prometaphase
8	NUF2	Kinetochore protein Nuf2	Acts as a component of the essential kinetochore-associated NDC80 complex, which is required for chromosome segregation and spindle checkpoint activity. Required for kinetochore integrity and the organization of stable microtubule binding sites in the outer plate of the kinetochore. The NDC80 complex synergistically enhances the affinity of the SKA1 complex for microtubules and may allow the NDC80 complex to track depolymerizing microtubules
9	MKI67	Proliferation marker protein Ki-67	Required to maintain individual mitotic chromosomes dispersed in the cytoplasm following nuclear envelope disassembly. Associates with the surface of the mitotic chromosome and the perichromosomal layer, and covers a substantial fraction of the chromosome surface. Prevents chromosomes from collapsing into a single chromatin mass by forming a steric and electrostatic charge barrier: the protein has a high net electrical charge and acts as a surfactant, dispersing chromosomes and enabling independent chromosome motility
10	BIRC5	Baculoviral IAP repeat containing 5	This gene is a member of the human CCDS set
11	CEP55	Centrosomal protein of 55 kDa	Plays a role in mitotic exit and cytokinesis. Recruits PDCD6IP and TSG101 to midbody during cytokinesis. Required for successful completion of cytokinesis. Not required for microtubule nucleation. Plays a role in the development of the brain and kidney
12	ANLN	Anillin	Required for cytokinesis. Essential for the structural integrity of the cleavage furrow and for completion of cleavage furrow ingression. Plays a role in bleb assembly during metaphase and anaphase of mitosis. May play a significant role in podocyte cell migration; pleckstrin homology domain containing

**Table 4:** Correlation analysis between NUF2 and immune cell-related genes and markers in TIMER.

Description	Gene markers	LUAD		LUSC	
		*R*	*P*	*R*	*P*
CD8+ T cell	CD8A	0.16	∗∗∗	0.018	0.689
	CD8B	0.200	∗∗∗	0.078	0.081
T cell (general)	CD3D	0.026	0.554	-0.065	0.144
	CD3E	-0.063	0.148	-0.140	∗∗∗
	CD2	-0.046	0.290	-0.080	0.075
B cell	CD19	-0.024	0.584	-0.180	∗∗∗
	CD79A	-0.040	0.360	-0.240	∗∗∗
Monocyte	CD86	0.024	0.573	-0.230	∗∗∗
	CSF1R	-0.17	∗∗∗	-0.350	∗∗∗
TAM	CCL2	-0.016	0.712	-0.150	∗∗
	CD68	-0.077	0.074	-0.270	∗∗∗
M1 macrophage	NOS2	-0.000	0.998	0.048	0.284
	IRF5	-0.025	0.56	0.004	0.930
	PTGS2	0.047	0.28	-0.230	∗∗∗
M2 macrophage	CD163	0.003	0.954	-0.290	∗∗∗
	VSIG4	-0.059	0.176	-0.23	∗∗∗
	MS4A4A	-0.058	0.181	-0.220	∗∗∗
Neutrophils	CEACAM8	-0.420	∗∗∗	-0.170	∗∗∗
	ITGAM	-0.058	∗∗∗	-0.32	∗∗∗
	CCR7	-0.220	∗∗∗	-0.210	∗∗∗
Natural killer cell	KIR2DL1	0.100	∗	-0.042	0.349
	KIR2DL3	0.150	∗∗∗	-0.007	0.88
	KIR2DL4	0.41	∗∗∗	0.017	0.712
	KIR3DL1	0.1	∗	-0.08	0.072
	KIR3DL2	0.120	∗∗	0.010	0.821
	KIR3DL3	0.240	∗∗∗	0.034	0.441
	KIR2DS4	0.1	∗	-0.053	0.239
Dendritic cell	HLA-DPB1	-0.390	∗∗∗	-0.280	∗∗∗
	HLA-DQB1	-0.350	∗∗∗	-0.220	∗∗∗
	HLA-DRA	-0.300	∗∗∗	-0.21	∗∗∗
	HLA-DPA1	-0.330	∗∗∗	-0.24	∗∗∗
	BDCA-1 (CD1C)	-0.510	∗∗∗	-0.310	∗∗∗
	BDCA-4 (NRP1)	-0.098	∗	-0.38	∗∗∗
	CD11c (ITGAX	-0.061	0.162	-0.270	∗∗∗
Th1	T-bet (TBX21)	-0.010	0.812	-0.083	0.064
	STAT4	-0.056	0.2	-0.270	∗∗∗
	STAT1	0.340	∗∗∗	0.038	0.398
	IFN-*γ* (IFNG)	0.310	∗∗∗	0.140	∗∗
	TNF-*α* (TNF)	-0.060	0.165	-0.220	∗∗∗
Th2	GATA3	-0.032	0.46	-0.280	∗∗∗
	STAT6	-0.360	∗∗∗	-0.130	∗∗
	STAT5A	-0.180	∗∗∗	-0.250	∗∗∗
	IL13	-0.029	0.507	-0.007	0.87
Tfh	BCL6	-0.160	∗∗∗	-0.027	0.544
	IL21	0.190	∗∗∗	0.003	0.954
Th17	STAT3	-0.21	∗∗∗	-0.200	∗∗∗
	IL17A	0.120	∗∗	0.069	0.124
Treg	FOXP3	0.009	0.836	-0.190	∗∗∗
	CCR8	0.011	0.807	-0.2	∗∗∗
	STAT5B	-0.12	∗∗	0.110	∗
	TGF*β* (TGFB1)	-0.260	∗∗∗	-0.430	∗∗∗
T cell exhaustion	PD-1 (PDCD1)	0.150	∗∗∗	-0.087	0.051
	CTLA4	0.11	∗∗	-0.072	0.109
	LAG3	0.230	∗∗∗	0.023	0.601
	TIM-3 (HAVCR2)	0.015	0.723	-0.180	∗∗∗
	GZMB	0.430	∗∗∗	0.032	0.480

^∗∗∗^ *P* < 0.001, ^∗∗^*P* < 0.01, and ^∗^*P* < 0.05.

**Table 5:** Clinical data of four cases of WB experiment.

	Gender	Age	Preoperative diagnosis	Pathological type
1	Male	69	Space-occupying lesion of upper right lung	Right lung squamous cell carcinoma
2	Male	68	Space-occupying lesion of right lower lung	Right lower lung squamous cell carcinoma
3	Female	73	Space-occupying lesion of right lower lung	Right lower lung adenocarcinoma
4	Male	71	Malignant tumor of left upper lung	Left upper lobe adenocarcinoma

## Data Availability

The data sets used and/or analyzed during the current study are available from the corresponding author on reasonable request.
